# *N*′-[(1*E*)-1-(2-Fluoro­phen­yl)ethyl­idene]pyridine-3-carbohydrazide

**DOI:** 10.1107/S1600536813035009

**Published:** 2014-01-08

**Authors:** P. B. Sreeja, M. Sithambaresan, N. Aiswarya, M. R. Prathapachandra Kurup

**Affiliations:** a Department of Chemistry, Christ University, Hosur Road, Bangalore 560 029, Karnataka, India; b Department of Chemistry, Faculty of Science, Eastern University, Sri Lanka, Chenkalady, Sri Lanka; c Department of Applied Chemistry, Cochin University of Science and Technology, Kochi 682 022, India

## Abstract

The title compound, C_14_H_12_FN_3_O, adopts an *E* conformation with respect to the azomethine double bond whereas the N and methyl C atoms are in a *Z* conformation with respect to the same bond. The ketonic O and azomethine N atoms are *cis* to each other. The non-planar mol­ecule [the dihedral angle between the benzene rings is 7.44 (11)°] exists in an amido form with a C=O bond length of 1.221 (2) Å. In the crystal, a bifurcated N—H⋯(O,N) hydrogen bond is formed between the amide H atom and the keto O and imine N atoms of an adjacent mol­ecule, leading to the formation of chains propagating along the *b*-axis direction. Through a 180° rotation of the fluoro­phenyl ring, the F atom is disordered over two sites with an occupancy ratio of 0.632 (4):0.368 (4).

## Related literature   

For biological properties of hydrazones, see: Sreeja *et al.* (2004 ▶); Ajani *et al.* (2010 ▶). For the synthesis of related compounds and for related work, see: Mangalam & Kurup (2011 ▶). For a related structure, see: Nair *et al.* (2012 ▶). For di­fluoro­phenyl compounds, see: Nayak *et al.* (2012 ▶).
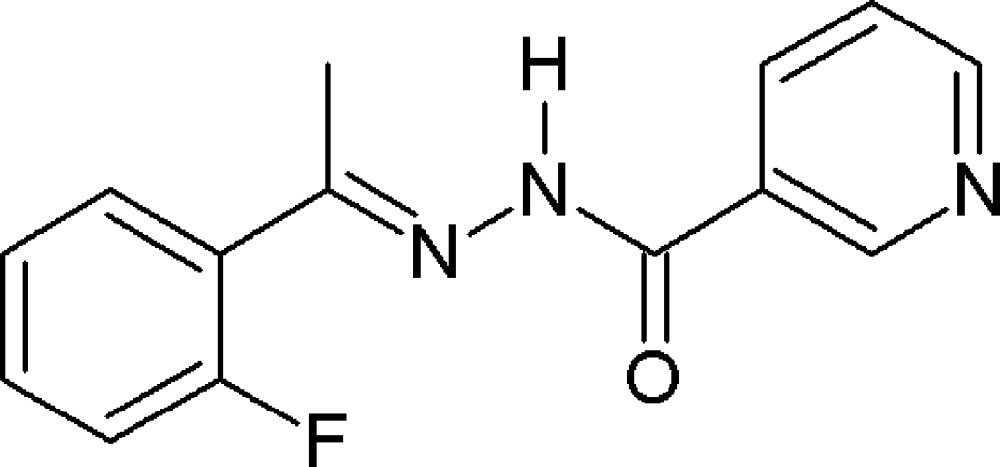


## Experimental   

### 

#### Crystal data  

C_14_H_12_FN_3_O
*M* *_r_* = 257.27
Orthorhombic, 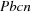

*a* = 18.926 (3) Å
*b* = 8.0486 (9) Å
*c* = 16.337 (3) Å
*V* = 2488.6 (7) Å^3^
*Z* = 8
Mo *K*α radiation
μ = 0.10 mm^−1^
*T* = 296 K
0.50 × 0.25 × 0.20 mm

#### Data collection  

Bruker ApexII CCD diffractometer
Absorption correction: multi-scan (*SADABS*; Bruker, 2004 ▶) *T*_min_ = 0.952, *T*_max_ = 0.980
19111 measured reflections
3009 independent reflections
1755 reflections with *I* > 2σ(*I*)
*R*_int_ = 0.056

#### Refinement  

*R*[*F* ^2^ > 2σ(*F* ^2^)] = 0.051
*wR*(*F* ^2^) = 0.161
*S* = 1.01
2999 reflections
188 parameters
1 restraint
H atoms treated by a mixture of independent and constrained refinement
Δρ_max_ = 0.29 e Å^−3^
Δρ_min_ = −0.22 e Å^−3^

### 

Data collection: *APEX2* (Bruker, 2004 ▶); cell refinement: *APEX2* and *SAINT* (Bruker, 2004 ▶); data reduction: *SAINT* and *XPREP* (Bruker, 2004 ▶); program(s) used to solve structure: *SHELXS97* (Sheldrick, 2008 ▶); program(s) used to refine structure: *SHELXL97* (Sheldrick, 2008 ▶); molecular graphics: *ORTEP-3 for Windows* (Farrugia, 2012 ▶) and *DIAMOND* (Brandenburg, 2010 ▶); software used to prepare material for publication: *SHELXL97* and *publCIF* (Westrip, 2010 ▶).

## Supplementary Material

Crystal structure: contains datablock(s) Global, I. DOI: 10.1107/S1600536813035009/zl2571sup1.cif

Structure factors: contains datablock(s) I. DOI: 10.1107/S1600536813035009/zl2571Isup2.hkl

Supporting information file. DOI: 10.1107/S1600536813035009/zl2571Isup3.cml

CCDC reference:

Additional supporting information:  crystallographic information; 3D view; checkCIF report

## Figures and Tables

**Table 1:** Hydrogen-bond geometry (Å, °)

*D*—H⋯*A*	*D*—H	H⋯*A*	*D*⋯*A*	*D*—H⋯*A*
N2—H2′⋯O1^i^	0.88 (1)	2.35 (1)	3.163 (2)	156 (2)
N2—H2′⋯N1^i^	0.88 (1)	2.48 (2)	3.148 (2)	133 (2)

Symmetry code: (i) 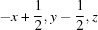.

**Fig. 1.**
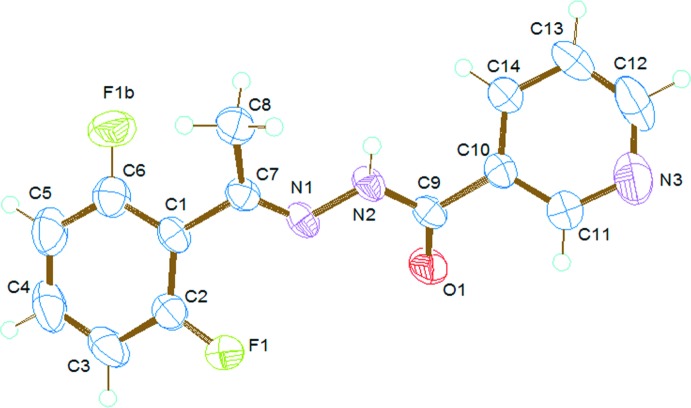
ORTEP diagram of N'-[(1E)-1-(2-fluorophenyl)ethylidene]pyridine-3-carbohydrazide with 50% probability ellipsoids. Disordered hydrogen atoms H2 and H6 were omitted for clarity.

**Fig. 2.**
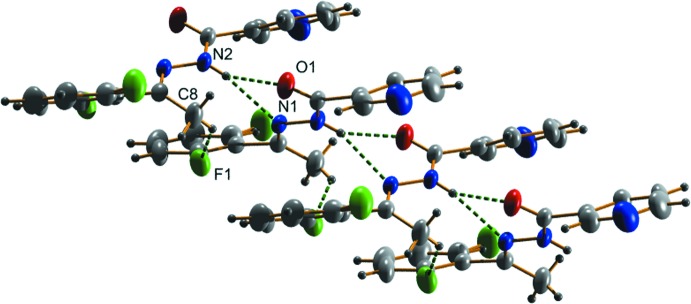
Bifurcated hydrogen-bonding interactions creating a chain propagating along the b-axis, and C—H···F interactions.

## supplementary crystallographic information

### 1. Comment

Hydrazones and their derivatives remain in the limelight of research due to the biological properties that many of them possess (Sreeja *et al.*, 2004; Ajani *et al.*, 2010). As a continuation of our work in this area (Mangalam & Kurup, 2011), we herein report a new aroyl hydrazone, derived from the reaction of 2-fluoroacetophenone and nicotinic acid hydrazide.

The molecule crystallizes in orthorhombic space group *P*bcn. The title compound adopts an *E* configuration with respect to the azomethine olefinic bond whilst the C8 and N2 atoms are in *Z* configuration with respect to the same bond with torsion angles for C1–C7–N1–N2 and N2–N1–C7–C8 of 177.76 (16) and -0.9 (3)°, respectively (Fig. 1). The ketonic O and the azomethine N are *cis* to each other with a torsion angle of -0.3 (3)°. The molecule exists in the amido form with a C9=O1 bond length of 1.221 (2) Å which is very close to the reported C=O bond length in a similar structure (Nair *et al.*, 2012). The pyridyl and fluorophenyl rings make dihedral angles of 45.44 (7) and 42.48 (6)° with the C(=O)N_2_CC central unit making the molecule non-planar.

A bifurcated hydrogen bond is formed between the amide H atom and the keto oxygen, O1 and imine nitrogen atom, N1 of an adjacent molecule which leads to formation of a hydrogen bonded chains of molecules that propagate along the b-axis direction of the structure (Fig. 2) with donor-acceptor distances of 3.163 (2) Å and 3.148 (2) Å respectively. In addition to the above intermolecular H bonding interactions, a non-classical H bond between H8A of methyl carbon C8 and the fluorine (F1) atom also stabilizes the packing. The non-classical H bonding features a C8···F1 distance of 3.169 (3) Å and links the molecules in a zigzag fashion (Fig. 2). No significant π···π interactions with centroid-centroid distances of less than 4 Å are observed in the structure.

Through a 180° rotation of the fluorophenyl ring, the fluorine atom F1 is disordered over two sites in a ratio of 63.3 (4):36.7 (4). Similar instances of positional disorder had been previously reported (Nayak *et al.*, 2012).

### 2. Experimental

The title compound was prepared by adapting a reported procedure (Mangalam & Kurup, 2011). Methanolic solutions of nicotinic acid hydrazide (0.137 g, 1 mmol) and 2-fluoroacetophenone (0.138 g, 1 mmol) were combined, a few drops of glacial acetic acid were added, and the mixture was heated to reflux for 6 h. On cooling the mixture, crystals of the hydrazone separated out. The crystals were filtered off, washed with a minimum quantity of methanol and dried over P_4_O_10_*in vacuo*. Crystals suitable for X-ray analysis were obtained in 80% yield (0.2048 g) from methanolic solution by slow evaporation. The melting point of the prepared compound was found to be 186 °C.

IR (KBr, υ in cm^-1^): 3269, 3058, 2858, 1673, 1621, 1539, 1487, 1356, 1273, 1179, 1121, 1063, 943, 799, 709, 651, 565, 513. ^1^H NMR (400 MHz, CDCl_3_, δ, p.p.m.): 10.95 (s, 1H), 7.26—9.03 (m, 9H), 2.36 (s, 3H).

### 3. Refinement

The disordered fluorine atoms F1 and F1B were refined freely, with the sum of their occupancy factors constrained to 1.0. Disordered hydrogen atoms H2 and H6 were placed in geometrically idealized positions and were constrained to ride on their parent C atoms with C–H distances of 0.93 Å. The N2—H2' distance was restrained to 0.88 (1) Å. The remaining H atoms were placed in calculated positions, guided by difference maps, with C–H bond distances 0.93–0.96 Å. H atoms were assigned as *U*_iso_(H) = 1.2*U*_eq_(carrier) or 1.5*U*_eq_(methyl C). Omitted owing to bad disagreement were the reflections (2 2 3), (5 2 1) and (2 0 0).

### Crystal data

**Table:**

C_14_H_12_FN_3_O	*F*(000) = 1072
*M**_r_* = 257.27	*D*_x_ = 1.373 Mg m^−^^3^
Orthorhombic, *P**b**c**n*	Mo *K*α radiation, λ = 0.71073 Å
Hall symbol: -P 2n 2ab	Cell parameters from 2433 reflections
*a* = 18.926 (3) Å	θ = 2.5–22.6°
*b* = 8.0486 (9) Å	µ = 0.10 mm^−^^1^
*c* = 16.337 (3) Å	*T* = 296 K
*V* = 2488.6 (7) Å^3^	Needle, colourless
*Z* = 8	0.50 × 0.25 × 0.20 mm

### Data collection

**Table:**

Bruker ApexII CCD diffractometer	3009 independent reflections
Radiation source: fine-focus sealed tube	1755 reflections with *I* > 2σ(*I*)
Graphite monochromator	*R*_int_ = 0.056
Detector resolution: 8.33 pixels mm^-1^	θ_max_ = 28.0°, θ_min_ = 2.5°
ω and φ scan	*h* = −24→25
Absorption correction: multi-scan (*SADABS*; Bruker, 2004)	*k* = −10→10
*T*_min_ = 0.952, *T*_max_ = 0.980	*l* = −21→21
19111 measured reflections	

### Refinement

**Table:**

Refinement on *F*^2^	Secondary atom site location: difference Fourier map
Least-squares matrix: full	Hydrogen site location: inferred from neighbouring sites
*R*[*F*^2^ > 2σ(*F*^2^)] = 0.051	H atoms treated by a mixture of independent and constrained refinement
*wR*(*F*^2^) = 0.161	*w* = 1/[σ^2^(*F*_o_^2^) + (0.0806*P*)^2^ + 0.3159*P*] where *P* = (*F*_o_^2^ + 2*F*_c_^2^)/3
*S* = 1.01	(Δ/σ)_max_ < 0.001
2999 reflections	Δρ_max_ = 0.29 e Å^−^^3^
188 parameters	Δρ_min_ = −0.22 e Å^−^^3^
1 restraint	Extinction correction: *SHELXL*, Fc^*^=kFc[1+0.001xFc^2^λ^3^/sin(2θ)]^-1/4^
Primary atom site location: structure-invariant direct methods	Extinction coefficient: 0.0048 (13)

### Special details

**Table:**

Geometry. All e.s.d.'s (except the e.s.d. in the dihedral angle between two l.s. planes) are estimated using the full covariance matrix. The cell e.s.d.'s are taken into account individually in the estimation of e.s.d.'s in distances, angles and torsion angles; correlations between e.s.d.'s in cell parameters are only used when they are defined by crystal symmetry. An approximate (isotropic) treatment of cell e.s.d.'s is used for estimating e.s.d.'s involving l.s. planes.
Refinement. Refinement of *F*^2^ against ALL reflections. The weighted *R*-factor *wR* and goodness of fit *S* are based on *F*^2^, conventional *R*-factors *R* are based on *F*, with *F* set to zero for negative *F*^2^. The threshold expression of *F*^2^ > σ(*F*^2^) is used only for calculating *R*-factors(gt) *etc*. and is not relevant to the choice of reflections for refinement. *R*-factors based on *F*^2^ are statistically about twice as large as those based on *F*, and *R*- factors based on ALL data will be even larger.

### Fractional atomic coordinates and isotropic or equivalent isotropic displacement parameters (Å^2^)

**Table:**

	*x*	*y*	*z*	*U*_iso_*/*U*_eq_	Occ. (<1)
O1	0.16369 (8)	1.08491 (17)	0.92895 (10)	0.0564 (5)	
N1	0.28297 (9)	1.04304 (17)	1.01301 (10)	0.0379 (4)	
N2	0.25250 (9)	0.91485 (18)	0.96943 (10)	0.0384 (4)	
N3	0.05671 (13)	0.6619 (3)	0.84194 (16)	0.0827 (8)	
F1	0.26405 (10)	1.2489 (2)	1.14815 (13)	0.0557 (7)	0.632 (4)
F1B	0.4820 (2)	1.1072 (5)	1.0493 (3)	0.0761 (16)	0.368 (4)
C6	0.44269 (12)	1.1919 (3)	1.09238 (15)	0.0533 (6)	
H6	0.4719	1.1180	1.0646	0.064*	0.632 (4)
C2	0.33059 (12)	1.2728 (2)	1.13857 (13)	0.0447 (5)	
H2	0.2822	1.2548	1.1430	0.054*	0.368 (4)
C5	0.47260 (14)	1.3288 (3)	1.12899 (17)	0.0672 (7)	
H5	0.5211	1.3466	1.1259	0.081*	
C4	0.43039 (15)	1.4381 (3)	1.16994 (18)	0.0717 (8)	
H4	0.4501	1.5317	1.1943	0.086*	
C3	0.35920 (15)	1.4110 (3)	1.17547 (16)	0.0628 (7)	
H3	0.3305	1.4850	1.2038	0.075*	
C1	0.37091 (10)	1.1596 (2)	1.09523 (12)	0.0378 (5)	
C7	0.33978 (10)	1.0136 (2)	1.05237 (12)	0.0371 (4)	
C8	0.37763 (12)	0.8504 (2)	1.05763 (16)	0.0551 (6)	
H8A	0.3441	0.7635	1.0681	0.083*	
H8B	0.4116	0.8544	1.1013	0.083*	
H8C	0.4015	0.8289	1.0069	0.083*	
C9	0.19212 (11)	0.9487 (2)	0.92867 (12)	0.0386 (5)	
C10	0.16141 (11)	0.8085 (2)	0.88031 (11)	0.0374 (5)	
C11	0.08970 (12)	0.7822 (3)	0.88351 (15)	0.0545 (6)	
H11	0.0626	0.8519	0.9164	0.065*	
C12	0.09829 (18)	0.5653 (4)	0.79477 (19)	0.0829 (9)	
H12	0.0769	0.4795	0.7658	0.099*	
C13	0.16935 (14)	0.5845 (3)	0.78654 (15)	0.0601 (6)	
H13	0.1954	0.5155	0.7522	0.072*	
C14	0.20124 (11)	0.7080 (2)	0.83009 (13)	0.0460 (5)	
H14	0.2498	0.7242	0.8259	0.055*	
H2'	0.2692 (10)	0.8137 (15)	0.9722 (14)	0.051 (6)*	

### Atomic displacement parameters (Å^2^)

**Table:**

	*U*^11^	*U*^22^	*U*^33^	*U*^12^	*U*^13^	*U*^23^
O1	0.0594 (10)	0.0345 (8)	0.0752 (12)	0.0116 (7)	−0.0128 (8)	−0.0112 (7)
N1	0.0495 (10)	0.0247 (7)	0.0393 (9)	−0.0057 (7)	−0.0023 (8)	−0.0022 (6)
N2	0.0499 (10)	0.0222 (7)	0.0431 (9)	−0.0004 (7)	−0.0070 (8)	−0.0031 (6)
N3	0.0724 (15)	0.0924 (17)	0.0834 (18)	−0.0245 (13)	−0.0065 (13)	−0.0225 (14)
F1	0.0434 (13)	0.0599 (13)	0.0639 (14)	0.0009 (9)	0.0058 (9)	−0.0186 (10)
F1B	0.053 (2)	0.072 (3)	0.103 (3)	−0.0039 (19)	0.026 (2)	−0.023 (2)
C6	0.0507 (13)	0.0508 (13)	0.0583 (14)	−0.0085 (11)	0.0020 (11)	−0.0027 (11)
C2	0.0508 (13)	0.0406 (11)	0.0426 (11)	0.0015 (9)	−0.0056 (10)	−0.0032 (8)
C5	0.0579 (15)	0.0559 (14)	0.088 (2)	−0.0180 (12)	−0.0146 (14)	−0.0041 (13)
C4	0.0798 (19)	0.0495 (14)	0.086 (2)	−0.0141 (13)	−0.0316 (16)	−0.0113 (13)
C3	0.0851 (19)	0.0412 (12)	0.0621 (15)	0.0095 (12)	−0.0157 (13)	−0.0182 (11)
C1	0.0479 (12)	0.0306 (9)	0.0347 (10)	−0.0059 (8)	−0.0019 (9)	0.0009 (7)
C7	0.0423 (11)	0.0307 (9)	0.0384 (10)	−0.0041 (8)	0.0034 (9)	−0.0009 (7)
C8	0.0528 (13)	0.0374 (11)	0.0751 (16)	0.0050 (10)	−0.0097 (12)	−0.0117 (10)
C9	0.0479 (11)	0.0262 (9)	0.0417 (11)	−0.0001 (8)	−0.0002 (9)	−0.0021 (7)
C10	0.0449 (11)	0.0305 (9)	0.0368 (10)	0.0001 (8)	−0.0059 (8)	0.0018 (7)
C11	0.0489 (13)	0.0561 (13)	0.0584 (15)	−0.0020 (11)	−0.0031 (11)	−0.0108 (11)
C12	0.106 (2)	0.0739 (18)	0.0687 (18)	−0.0330 (18)	−0.0120 (17)	−0.0239 (15)
C13	0.0832 (18)	0.0477 (12)	0.0492 (13)	−0.0034 (12)	0.0003 (12)	−0.0194 (10)
C14	0.0496 (12)	0.0428 (11)	0.0457 (12)	−0.0013 (9)	−0.0019 (10)	−0.0070 (9)

### Geometric parameters (Å, º)

**Table:**

O1—C9	1.221 (2)	C4—C3	1.368 (4)
N1—C7	1.275 (2)	C4—H4	0.9300
N1—N2	1.380 (2)	C3—H3	0.9300
N2—C9	1.350 (3)	C1—C7	1.490 (3)
N2—H2'	0.875 (9)	C7—C8	1.498 (3)
N3—C11	1.338 (3)	C8—H8A	0.9600
N3—C12	1.348 (4)	C8—H8B	0.9600
F1—C2	1.284 (3)	C8—H8C	0.9600
F1B—C6	1.230 (4)	C9—C10	1.495 (3)
C6—C5	1.376 (3)	C10—C11	1.375 (3)
C6—C1	1.384 (3)	C10—C14	1.377 (3)
C6—H6	0.9300	C11—H11	0.9300
C2—C3	1.376 (3)	C12—C13	1.360 (4)
C2—C1	1.383 (3)	C12—H12	0.9300
C2—H2	0.9300	C13—C14	1.363 (3)
C5—C4	1.364 (4)	C13—H13	0.9300
C5—H5	0.9300	C14—H14	0.9300
			
C7—N1—N2	118.26 (15)	C6—C1—C7	121.36 (18)
C9—N2—N1	117.22 (15)	N1—C7—C1	115.06 (16)
C9—N2—H2'	121.3 (14)	N1—C7—C8	126.53 (17)
N1—N2—H2'	121.2 (14)	C1—C7—C8	118.40 (17)
C11—N3—C12	115.8 (2)	C7—C8—H8A	109.5
F1B—C6—C5	116.3 (3)	C7—C8—H8B	109.5
F1B—C6—C1	120.6 (3)	H8A—C8—H8B	109.5
C5—C6—C1	122.7 (2)	C7—C8—H8C	109.5
F1B—C6—H6	6.8	H8A—C8—H8C	109.5
C5—C6—H6	118.7	H8B—C8—H8C	109.5
C1—C6—H6	118.7	O1—C9—N2	123.50 (17)
F1—C2—C3	117.0 (2)	O1—C9—C10	120.55 (18)
F1—C2—C1	120.32 (19)	N2—C9—C10	115.94 (15)
C3—C2—C1	122.6 (2)	C11—C10—C14	118.21 (19)
F1—C2—H2	2.6	C11—C10—C9	118.68 (18)
C3—C2—H2	118.7	C14—C10—C9	123.06 (18)
C1—C2—H2	118.7	N3—C11—C10	123.6 (2)
C4—C5—C6	119.3 (2)	N3—C11—H11	118.2
C4—C5—H5	120.4	C10—C11—H11	118.2
C6—C5—H5	120.4	N3—C12—C13	124.6 (2)
C5—C4—C3	120.4 (2)	N3—C12—H12	117.7
C5—C4—H4	119.8	C13—C12—H12	117.7
C3—C4—H4	119.8	C12—C13—C14	118.0 (2)
C4—C3—C2	119.2 (2)	C12—C13—H13	121.0
C4—C3—H3	120.4	C14—C13—H13	121.0
C2—C3—H3	120.4	C13—C14—C10	119.8 (2)
C2—C1—C6	115.79 (18)	C13—C14—H14	120.1
C2—C1—C7	122.84 (18)	C10—C14—H14	120.1
			
C7—N1—N2—C9	179.58 (18)	C6—C1—C7—N1	−135.9 (2)
F1B—C6—C5—C4	172.7 (4)	C2—C1—C7—C8	−138.5 (2)
C1—C6—C5—C4	0.0 (4)	C6—C1—C7—C8	42.9 (3)
C6—C5—C4—C3	0.8 (4)	N1—N2—C9—O1	−0.3 (3)
C5—C4—C3—C2	−0.6 (4)	N1—N2—C9—C10	178.48 (16)
F1—C2—C3—C4	177.3 (2)	O1—C9—C10—C11	−44.3 (3)
C1—C2—C3—C4	−0.5 (4)	N2—C9—C10—C11	136.9 (2)
F1—C2—C1—C6	−176.5 (2)	O1—C9—C10—C14	133.2 (2)
C3—C2—C1—C6	1.2 (3)	N2—C9—C10—C14	−45.7 (3)
F1—C2—C1—C7	4.8 (3)	C12—N3—C11—C10	−0.5 (4)
C3—C2—C1—C7	−177.5 (2)	C14—C10—C11—N3	1.6 (3)
F1B—C6—C1—C2	−173.4 (3)	C9—C10—C11—N3	179.1 (2)
C5—C6—C1—C2	−1.0 (3)	C11—N3—C12—C13	−1.0 (5)
F1B—C6—C1—C7	5.3 (4)	N3—C12—C13—C14	1.3 (5)
C5—C6—C1—C7	177.7 (2)	C12—C13—C14—C10	−0.1 (4)
N2—N1—C7—C1	177.76 (16)	C11—C10—C14—C13	−1.2 (3)
N2—N1—C7—C8	−0.9 (3)	C9—C10—C14—C13	−178.7 (2)
C2—C1—C7—N1	42.7 (3)		

### Hydrogen-bond geometry (Å, º)

**Table:**

*D*—H···*A*	*D*—H	H···*A*	*D*···*A*	*D*—H···*A*
N2—H2′···O1^i^	0.88 (1)	2.35 (1)	3.163 (2)	156 (2)
N2—H2′···N1^i^	0.88 (1)	2.48 (2)	3.148 (2)	133 (2)

Symmetry code: (i) −*x*+1/2, *y*−1/2, *z*.
